# In‐Memory‐Computed Low‐Frequency Noise Spectroscopy for Selective Gas Detection Using a Reducible Metal Oxide

**DOI:** 10.1002/advs.202205725

**Published:** 2023-01-16

**Authors:** Wonjun Shin, Jaehyeon Kim, Gyuweon Jung, Suyeon Ju, Sung‐Ho Park, Yujeong Jeong, Seongbin Hong, Ryun‐Han Koo, Yeongheon Yang, Jae‐Joon Kim, Seungwu Han, Jong‐Ho Lee

**Affiliations:** ^1^ Department of Electrical and Computer Engineering and Inter‐university Semiconductor Research Center Seoul National University Seoul 08826 Republic of Korea; ^2^ Department of Materials Science and Engineering and Research Institute of Advanced Materials Seoul National University Seoul 08826 Republic of Korea; ^3^ Research and Development Division SK Hynix Inc. Icheon 17736 Republic of Korea; ^4^ Ministry of Science and ICT Sejong 30121 Republic of Korea

**Keywords:** in‐memory‐computing, low‐frequency noise (LFN), selective detection, tungsten oxide

## Abstract

Concerns about indoor and outdoor air quality, industrial gas leaks, and medical diagnostics are driving the demand for high‐performance gas sensors. Owing to their structural variety and large surface area, reducible metal oxides hold great promise for constructing a gas‐sensing system. While many earlier reports have successfully obtained a sufficient response to various types of target gases, the selective detection of target gases remains challenging. In this work, a novel method, low‐frequency noise (LFN) spectroscopy is presented, to achieve selective detection using a single FET‐type gas sensor. The LFN of the sensor is accurately modeled by considering the charge fluctuation in both the sensing material and the FET channel. Exposure to different target gases produces distinct corner frequencies of the power spectral density that can be used to achieve selective detection. In addition, a 3D vertical‐NAND flash array is used with the fast Fourier transform method via in‐memory‐computing, significantly improving the area and power efficiency rate. The proposed system provides a novel and efficient method capable of selectively detecting a target gas using in‐memory‐computed LFN spectroscopy and thus paving the way for the further development in gas sensing systems.

## Graphical abstract

The fundamental obstacle faced by metal oxide‐based gas sensors is the cross‐sensitivity among different gases. Low‐frequency noise (LFN) spectroscopy in horizontal floating‐gate FET‐type gas sensors is presented to address this issue. The proposed LFN spectroscopy is implemented using in‐memory‐computed FFT computations based on 3D V‐NAND for an area‐ and energy‐efficient operations.

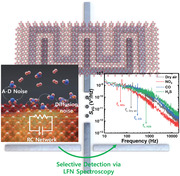

## 1 Introduction

Recent years have witnessed a boost in gas sensor research and development as there are increasing demands for high‐performance gas sensors in various fields.^[^ ^1^, ^2^, ^3^, ^4^ ^]^ Rapid industrialization and urbanization have led to an increase in air pollution caused by the combustion of fossil fuels and emissions from a variety of sources, including automobiles, electricity generation, agricultural waste incineration, and factories.^[^ ^5^, ^6^, ^7^ ^]^ In this regard, it is vital to collect location‐ and time‐specific data on gas emissions so as to monitor daily pollution levels.^[^ ^8^, ^9^ ^]^ Reducible metal oxide‐based gas sensors are the most viable choice for the actuation of a reliable gas sensing system due to their large response to various gases, simple fabrication method, ease of operation, and low fabrication cost.^[^ ^10^, ^11^, ^12^ ^]^ Accordingly, there have been extensive studies to improve the sensing capabilities of metal oxide‐based gas sensors. Because it is crucial to detect a low concentration of gas stably in many applications, studies to improve a limit‐of‐detection (LOD) of sensors to the target gas have been intensively conducted in both academia and industry.^[^ ^12^ ^]^

In sensor applications, the LOD is determined by the signal‐to‐noise ratio (SNR) of the sensor. Therefore, most previous studies focused on increasing the response (signal) and thus the SNR and LOD, including the introduction of new sensing materials^[^ ^13^, ^14^, ^15^ ^]^ or modification of the morphologies of the sensing materials to the nanostructures.^[^ ^16^, ^17^, ^18^ ^]^ On the other hand, the noise of the sensor is generally considered to be an unfavorable element that degrades the SNR. Therefore, it should be minimized and ultimately eliminated.^[^ ^19^, ^20^, ^21^, ^22^, ^23^ ^]^ As a result, previous studies have concentrated on minimizing the noise or engineering a trade‐off relationship between the response and noise to improve the SNR. However, recently, in contrast to conventional approaches that consider noise a nuisance and attempt to minimize it, various applications have been proposed that utilize the noise of electronic devices. In sensor applications, the noise spectrum of the output sensing signal can be used as a unique sensing feature to identify a target gas selectively, a technique known as selective detection using low‐frequency noise (LFN) spectroscopy.^[^ ^24^, ^25^, ^26^ ^]^ LFN spectroscopy makes use of changes in the shape of the power spectral density (PSD) of the sensor before and after the gas reaction. Ruyantsev et al. reported that the corner frequency (*f*_c_) at which the Lorentzian bulge occurs varies depending on the type of gas.^[^ ^24^ ^]^ Different *f*_c_s depending on gas species can be used as a parameter to achieve selective detection. LFN spectroscopy has a significant advantage over other methods in that selective detection can be accomplished with a single sensor, whereas other methods require a dense sensor array to collect various sensing data.

Selective detection based on LFN spectroscopy was only realized in two‐terminal resistor‐type gas sensors in previous studies.^[^ ^24^, ^25^, ^26^ ^]^ Although resistor‐type gas sensors have the advantages of a simple fabrication process and large response, they also undergo device‐to‐device variations, making it difficult to ensure the reliability of the sensing system.^[^ ^10^ ^]^ In particular, the variability of the resistor‐type gas sensor is a substantial impediment to realizing selective detection using LFN spectroscopy. In contrast, FET‐type gas sensors with a horizontal floating‐gate (FG) have higher integration degrees and much better reliability.^[^ ^27^, ^28^, ^29^, ^30^ ^]^ Therefore, it is crucial to realize selectivity by utilizing LFN spectroscopy with a FET‐type sensor. However, it is regarded that the LFN characteristics of FET‐type gas sensors are determined by the FET transducer, not by the sensing materials,^[^ ^27^, ^28^ ^]^ which makes it difficult to utilize LFN spectroscopy for selective detection. However, these characteristics can vary depending on the type or structure of the sensing material. Therefore, an accurate noise model for a FET‐type gas sensor considering the charge fluctuations in both the sensing material and the FET transducer should be provided.

Building an area‐ and energy‐efficient system is critical when attempting to apply LFN spectroscopy to detect a target gas selectively. Due to the massive number of multiplication and accumulation (MAC) operations required by the Fast Fourier Transform (FFT) for PSD acquisition, energy consumption is inevitably high when adopting the conventional von Neumann computing architecture.^[^ ^31^ ^]^ Particularly, in the von Neumann architecture, the physical and operational separation between the memory and the processor units is the primary reason for the significant latency and power consumption when performing MAC operations. In recent years, in‐memory computing (IMC) has emerged as a new computing paradigm that reduces data movement between the memory and processing units by adopting massive parallelism. The MAC burden when computing the FFT can also be effectively reduced by adopting IMC.^[^ ^32^ ^]^ Constructing a gas sensing system using IMC‐based LFN spectroscopy is therefore appealing from an area and energy efficiency standpoint.

Following the above discussion, in this study, we propose and experimentally demonstrate a novel gas sensing system, IMC LFN spectroscopy, to realize selective detection among various gases based on a FET‐type gas sensor with horizontal FG. Tungsten‐oxide (WO_3_) is used as a sensing material and is deposited between the control‐gate (CG) and the FG. The LFN characteristics of FET‐type gas sensors with a WO_3_ sensing layer are systematically investigated, and an accurate noise model for the FET‐type gas sensor is proposed, including the effects of charge fluctuation in the sensing area and FET transducer. In addition, the effects of external stimuli, including the temperature and electrical bias, on the LFN characteristics are investigated to confirm the validity of the proposed noise model, and the LFN characteristics of FET‐type gas sensors with different sensing materials (indium‐oxide (In_2_O_3_), indium‐gallium‐zinc‐oxide (IGZO), and vanadium‐oxide (V_2_O_5_)) are systematically investigated and compared, thus expanding the knowledge of the LFN behaviors of gas sensors. It is verified through density functional theory (DFT) calculations that WO_3_ is the most suitable material for LFN spectroscopy in FET‐type sensors. Finally, we experimentally realize an in‐memory‐computed FFT system for LFN spectroscopy using a 3D vertical‐NAND (V‐NAND) flash array. The proposed LFN spectroscopy for selective detection has merits in terms of reliability and energy efficiency compared to previous studies that adopt nanostructured sensing materials^[^ ^33^ ^]^ or a dense array of sensors, which requires complicated data processing.^[^ ^34^ ^]^ The results in this study pave the way for the building of area‐ and energy‐efficient gas sensing systems for selective detection with excellent reliability.

## 2 Results and Discussion

### 2.1 System Architecture

**Figure 1:**
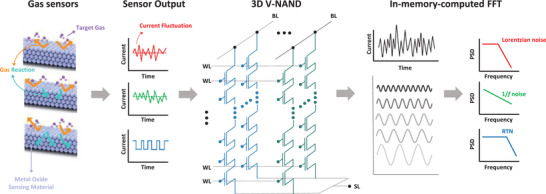
Schematic diagram of the proposed gas sensing system for in‐memory‐computed LFN spectroscopy. The sensing process is as follows: 1) The target gas is exposed to the FET‐type gas sensor with a horizontal FG having reducible WO_3_ as a sensing material. 2) The output signal of the sensor (*I*_D_) is fed into 3D V‐NAND for computing the FFT. 3) The PSD of the output signal is obtained for selective detection using LFN spectroscopy. The PSD of the output signal varies depending on the gas type, and the variation can be used as a unique sensing feature for selective detection.

**Figure** **1** shows a schematic diagram of the proposed system for in‐memory‐computed LFN spectroscopy to detect a target gas selectively. When a reducible metal oxide interacts with the target gas, the drain current (*I*_D_) of the FET‐type gas sensor is changed, serving as a sensing signal. In general, fluctuation of the output signal is considered to be a detrimental factor that degrades the SNR. However, in this study, we propose a method to utilize these current fluctuations as a unique sensing feature that can be used for selective gas detection. The current fluctuations show different behaviors depending on type of gas, and these can be analyzed by LFN spectroscopy. Thus, the output currents of the sensor are fed into the 3D vertical‐NAND (V‐NAND) array that implements the Fast Fourier Transform (FFT) in an IMC manner. The technologically mature 3D V‐NAND flash array implements FFT for LFN spectroscopy with excellent energy and area efficiency. Note that the V‐NAND flash memory in this work is industrially fabricated with the help of SK Hynix.

### 2.2 Sensor Structure

**Figure 2:**
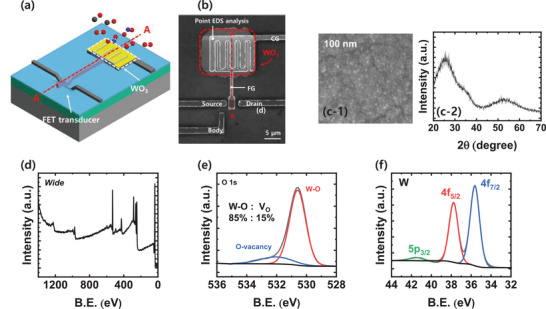
a) 3‐D schematic bird's‐eye view of a FET‐type gas sensor with a horizontal FG. b) Top scanning electron microscopy (SEM) image of the fabricated sensor. c‐1) Top SEM image and c‐2) GIXRD of the WO_3_ thin film, respectively. d) Wide‐scan XPS spectrum, high resolution XPS spectra of e) O 1s and f) W, respectively.

The FET‐type gas sensor with a horizontal FG is used as a sensor platform, and a reducible metal oxide (WO_3_) is used as a sensing layer for selective gas detection. **Figure** **2**a,b shows a 3D schematic bird's eye view and top scanning electron microscope (SEM) image of the FET‐type gas sensor, respectively. The detailed fabrication process of the sensor is described in Figure S1 (Supporting Information). The FET transducer for the sensor has a channel width/length (*W*/*L*) of 2 µm/2 µm.

A 20 nm‐thick *n*‐type semiconducting WO_3_ film is deposited by the sputtering method at 20 °C. Figure 2c(1,2) correspondingly shows a top SEM image and the results of a grazing incidence X‐ray diffraction analysis (GIXRD) of WO_3_ deposited between the CG and FG. These figures show that deposited film is amorphous. A wide‐scan X‐ray photoelectron spectroscopy (XPS) spectrum and high‐resolution XPS spectra of O 1s and W 4f are shown in Figure 2d–f, respectively. In a quantitative analysis based on the area of the spectra, the W:O composition ratio is set to 1:3. In the O 1s XPS spectrum, the binding energies (B.E.s) of 530.26 and 532.01 eV correspond to W–O bonding and oxygen vacancies. In the W XPS spectrum, the B.E.s of 34.27 and 36.45 eV correspond to W^5+^ 4f_7/2_ and W^5+^ 4f^5/2^, respectively.

### 2.3 LFN Characteristics of the FET‐Type Gas Sensor with a WO_3_ Sensing Layer

**Figure 3:**
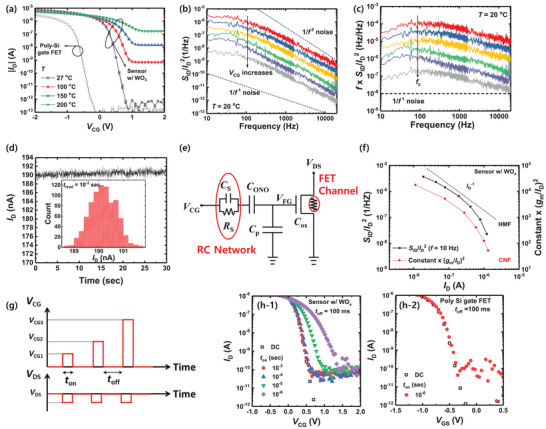
a) Transfer characteristics (*I*_D_‐*V*_CG_) of a FET‐type gas sensor with a WO_3_ sensing depending on the temperature (*T*). The *I*_D_‐*V*_CG_ of the poly‐Si gate FET used as a sensor platform is indicated by the open symbols. b) *S*_ID_/*I*_D_^2^ and c) *f*  × *S*_ID_/*I*_D_^2^ as a function of *f* under different bias conditions. Here, *V*_DS_ is set to −0.1 V and *V*_CG_ ranges from −0.5 to 0.5 V. d) *I*_D_ variation over time in the bias condition where Lorentzian‐like noise is observed (*I*_D_ = 190 nA). The inset shows the corresponding *I*_D_ amplitude distribution. e) Equivalent circuit diagram of the FET‐type gas sensor. *C*_S_, *C*_ONO_, *C*_ox_, and *C*_P_ denote the capacitances of the sensing layer, the O/N/O layer, the gate oxide, and the parasitic capacitance, respectively. *R*_S_ is the resistance of the sensing material. f) *S*_ID_/*I*_D_^2^ sampled at 10 Hz and (*g*_m_/*I*_D_)^2^ multiplied by a constant versus *I*_D_ of the sensor. g) Bias scheme for the PIV measurement. *I*_D_‐*V*_CG_ and *I*_D_‐*V*_GS_ of the h‐1) FET‐type gas sensor and h‐2) poly‐Si gate FET with DC and PIV measurements at various *t*_on_ values, respectively.

**Figure** **3**a shows the transfer characteristics (*I*_D_‐*V*_CG_) of the FET‐type gas sensor with a WO_3_ sensing layer depending on the temperature (*T*). The sensor shows typical *p*‐type FET characteristics with a threshold voltage (*V*_th_) of 0.28 V and a subthreshold swing of 90 mV at 27 °C. Open symbols represent the *I*_D_‐*V*_CG_ curves measured at 27 °C for the poly‐Si gate FET used here as the sensor platform. The *V*_th_ of the sensor with WO_3_ is smaller than that of the poly‐Si gate FET due to the work function difference between the poly‐Si and WO_3_. The off‐current increases with an increase in *T* as the source/drain‐to‐substrate junction leakage current increases. The slight decrease in the on‐current with the increase in *T* is due to the increased carrier scattering in the FET channel.

Figure 3b shows the normalized drain current noise PSD (*S*_ID_/*I*_D_^2^) as a function of the frequency (*f*) under different bias conditions. *V*_DS_ is set to −0.1 V, and *V*_CG_ ranges from −0.5 to 0.5 V. The PSDs are measured at *T* = 27 °C. In previous studies, it was reported that the LFN characteristics of FET‐type gas sensors are determined by FET transducers.^[^ ^27^, ^28^, ^29^ ^]^ When an *n*‐type FET with a surface channel structure is adopted as a sensor platform, the LFN characteristics of the sensor are determined by the carrier number fluctuation (CNF) at the gate oxide‐FET channel interface.^[^ ^29^ ^]^ On the other hand, the carrier mobility fluctuation (CMF) in the Si bulk determines the LFN characteristics when a *p*‐type FET with a buried channel structure is adopted as a sensor platform.^[^ ^27^, ^28^ ^]^ Such features are explained by Hooge's mobility fluctuation (HMF) model.^[^ ^27^, ^33^ ^]^ In both cases, 1/*f ^*γ*^* noise with *γ* ∼ 1 is dominant in the low‐frequency domain. However, the FET‐type gas sensor with the WO_3_ sensing layer shows a Lorentzian curve with a distinct *f*_c_ at *f* = 100 Hz, as shown in Figure 3c. Note that if the sensor shows 1/*f* noise behavior, *f*  × PSD should be constant regardless of the change in *f*, as denoted by the dashed line in Figure 3c.

In general, Lorentzian noise stems from random telegraph noise (RTN) generated from the trapping/detrapping process of the carriers in the FET channel to/from traps inside a gate oxide. However, the Lorentzian noise behavior of the FET‐type gas sensor cannot be explained by RTN, and the reasons can be summarized as follows: 1) The FET transducer has a *p*‐type FET with a buried channel structure whose conductive channel is formed ≈20 nm away from the gate oxide (Figure S2a, Supporting Information). The inset of Figure S2a (Supporting Information) shows the schematics of the FET channel in the poly‐Si gate FET. Figure S2b (Supporting Information) shows the *S*_ID_/*I*_D_^2^ of the poly‐Si gate FET as a function of *f* under various bias conditions. Figure S2c (Supporting Information) shows the *S*_ID_/*I*_D_^2^ and constant ×  (*g*_m_/*I*_D_)^2^ versus *I*_D_ of the poly‐Si gate FET. The behavior of *S*_ID_/*I*_D_^2^ deviates from that of (*g*_m_/*I*_D_)^2^ with respect to *I*_D_, verifying that the LFN characteristics of the poly‐Si gate FET do not stem from the CNF. Rather, the slope of *S*_ID_/*I*_D_^2^ with respect to *I*_D_ is ‐1, indicating that the 1/*f* noise originates from the CMF in the silicon substrate.^[^ ^27^ ^]^ Therefore, it is not plausible that the trapping/detrapping process of carriers to/from oxide traps can be the main factor that determines the overall LFN characteristics of the sensor. 2) The trapping/detrapping processes of the carriers to/from traps inside a gate oxide are strongly affected by the gate bias; and accordingly, *f*_c_ shows bias dependence.^[^ ^34^, ^35^ ^]^ However, the *f*_c_ of the FET‐type gas sensor does not change with an increase in *V*_CG_ (Figure 3b,c). 3) If trapping/detrapping processes at a certain defect dominate the LFN characteristics, trap occupancy switching results in a discrete current fluctuation (two or multi‐level RTN).^[^ ^36^, ^37^, ^38^ ^]^ However, a Gaussian distribution of the current amplitude is observed in the sensor. Figure 3d shows the variation of *I*_D_ over time in the bias condition where the Lorentzian‐like noise is observed. Here, *I*_D_ does not show discrete current fluctuations, as verified by the corresponding *I*_D_ amplitude distribution (Inset of Figure 3d). In addition, *I*_D_ shows a Gaussian distribution. Figure S3 (Supporting Information) shows the Gaussian distribution of three different *I*_D_s. Therefore, it can be concluded that the Lorentzian‐like noise of the sensor does not stem from the trapping/detrapping process in the FET channel.

In FET‐type gas sensors, the sensing material deposited between the CG and FG can also be considered as a possible noise source. Figure 3e shows an equivalent circuit diagram of a FET‐type gas sensor. *C*_S_, *C*_ONO_, *C*_ox_, and *C*_P_ denote the capacitances of the sensing layer, the O/N/O layer, the gate oxide, and the parasitic capacitance, respectively. *R*_S_ is the resistance of the sensing material. If there are charge fluctuations in the sensing material between the CG and FG, the voltage coupled between the CG and FG fluctuates, in turn causing *I*_D_ to fluctuate. There are two main sources that can contribute to the charge fluctuation in the sensing material: 1) Number fluctuation of the adsorbed gas and 2) Charge fluctuation due to the carrier exchange between the sensing material and the adsorbed gas.

1) First, we consider the number fluctuation of the adsorbed gas, the behavior of which can be understood with an analogy of the generation‐recombination (G‐R) noise.^[^ ^39^, ^40^ ^]^ The adsorption and desorption of gas on the sensing material are governed by the adsorption (*τ*_ads_) and the desorption time (*τ*_des_). The PSD of the gate voltage due to the adsorption and desorption can be derived by following the procedure used for the G‐R noise.
(1) $$\bar{\Delta V_{g,\mathrm{sensing}1}^{2}\left(f\right)}=\frac{4V_{m}^{2}\bar{\Delta N_{\mathrm{ads}}^{2}}\tau_{1}^{2}}{\tau_{\mathrm{des}}\left[1+{\left(2\pi f\tau \right)}^{2}\right]}$$
  with
(2) $$\frac{1}{\tau }=\frac{1}{\tau_{\mathrm{ads}}}+\frac{1}{\tau_{\mathrm{des}}}$$
  where *V*_m_ is the effective gate voltage due to a single adsorbed molecule and Δ*N*_ads_ is the fluctuation of the number of adsorbed molecules. In this case, the PSD shows a Lorentzian curve with a *f*_c_ of 1/*τ*. However, considering that the adsorption/desorption process of gas molecules to/from the sensing material is very slow, the PSD has a very low *f*_c_. It has been reported that a typical value of *f*_c_, is ≈10^−2^ Hz.^[^ ^24^, ^26^ ^]^ Therefore, it is difficult to explain the LFN characteristics of these types of sensors with the adsorption/desorption noise of gas molecules.
2) Another factor to be considered is the charge fluctuation generated from the carrier exchange between the sensing material and the adsorbed molecules. During the carrier exchange process, a local diffusion current is formed. The corresponding PSD can be modeled as shown below^[^ ^41^ ^]^
(3) $$\bar{\Delta I_{g,\mathrm{sensing}2}^{2}}=\frac{4kT}{R_{S}}.$$

As shown in Figure 3e, a resistor‐capacitor (RC) network composed of a parallel combination of *R*_S_ and *C*_S_ exists in the sensing material. The local current noise can be transferred to the voltage noise by the transfer function of the RC network. The transfer function is expressed as

(4) $$H\left(s\right)=\frac{1}{1+sR_{S}C_{\mathrm{eq}}}$$

where

(5) $$C_{\mathrm{eq}}=C_{S}+C_{p}+C_{\mathrm{ONO}}$$

Thus, the voltage noise PSD ($\bar{\Delta V_{g,\mathrm{sensing}2}^{2}}$) is given by

(6) $$\bar{\Delta V_{g,\mathrm{sensing}2}^{2}\left(f\right)}={\left|H\left(s\right)\right|}^{2}\bar{\Delta I_{g,\mathrm{sensing}2}^{2}}=\frac{4kTR_{S}}{1+{\left(2\pi R_{S}C_{\mathrm{eq}}f\right)}^{2}}.$$

Accordingly, $\bar{\Delta V_{g,\mathrm{sensing}2}^{2}}$ has the shape of a low‐pass filter with a corner frequency (*f*_c,2_) of 1/*R*_S_ *C*_eq_. Because $\bar{\Delta V_{g,\mathrm{sensing}1}^{2}\left(f\right)}$ and $\bar{\Delta V_{g,\mathrm{sensing}2}^{2}\left(f\right)}$ are uncorrelated, the total noise PSD in the sensing material ($\bar{\Delta V_{g}^{2}\left(f\right)}$) is the superposition of $\bar{\Delta V_{g,1}^{2}\left(f\right)}$ and $\bar{\Delta V_{g,2}^{2}\left(f\right)}$, which can be expressed as

(7) $$\bar{\Delta V_{g}^{2}\left(f\right)}=\bar{\Delta V_{g,\mathrm{sensing}1}^{2}\left(f\right)}+\bar{\Delta V_{g,\mathrm{sensing}2}^{2}\left(f\right)}.$$

When $\bar{\Delta V_{g,\mathrm{sensing}}^{2}\left(f\right)}$ is reflected in the drain current fluctuation, its amplitude is multiplied by *g*_m_^2^. Accordingly, the drain current spectral density generated from the sensing area (*S*_ID,sensing_) is expressed as

(8) $$S_{\mathrm{ID},\mathrm{sensing}}=g_{m}^{2}\bar{\Delta V_{g}^{2}\left(f\right)}$$

Figure 3f shows *S*_ID_/*I*_D_^2^ sampled at 10 Hz and (*g*_m_/*I*_D_)^2^ multiplied by the constant versus *I*_D_ of the sensor. Here, *S*_ID_/*I*_D_^2^ and (*g*_m_/*I*_D_)^2^ show the same behavior with respect to *I*_D_, verifying that Equation 8 is well fitted to the noise of the sensor.

In summary, the total *S*_ID_ of the sensor is the result of the superposition of the FET intrinsic 1/*f* noise (*S*_ID,FET_) and *S*_ID,sensing_:

(9) $$S_{\mathrm{ID}}=S_{\mathrm{ID},\mathrm{sensing}}+S_{\mathrm{ID},\mathrm{FET}}$$

The reason why *S*_ID_ shows Lorentzian‐like behavior in a measured frequency range is that the magnitude of *S*_ID,sensing_ is larger than that of the FET intrinsic 1/*f* noise. Indeed the measured *S*_ID_/*I*_D_^2^ is much larger than the *S*_ID,FET_/*I*_D,FET_^2^ (see Figure 3b). Figure S4 (Supporting Information) explains how *S*_ID_ is determined from the interplay between *S*_ID,sensing_ and *S*_ID,FET_ in the frequency domain.

To verify the existence of the RC network in the sensing material region, we take pulsed *I*–*V* (PIV) measurements of the FET‐type gas sensor and compare these outcomes with those of a poly‐Si gate FET. It should be noted that the WGFMU module is used for the PIV measurement. Figure 3g shows the bias scheme used for the PIV measurement. Figure 3h‐1 shows the *I*_D_‐*V*_CG_ of the FET‐type gas sensor with DC and pulsed *I*–*V* (PIV) measurements at various values of *t*_on_. The off‐current of the FET‐type gas sensor as determined by the PIV measurement is much larger than that by the DC measurement. This occurs due to the lower limit of the WGFMU module in the current measurement (100 pA). With a decrease in *t*_on_, the subthreshold swing (SS) increases due to the RC delay caused by the RC network in the sensing area. Such a delay in the *I*_D_‐*V*_GS_ curves measured by PIV is in accordance with the LFN measurement results. Contrary to the FET‐type gas sensor, the poly‐Si gate FET does not show an increase in SS, even at a *t*_on_ value of 10^−6^ s, as shown in Figure 3h‐2. These results confirm that the RC network of the FET‐type gas sensor stems from the sensing material.

**Figure 4:**
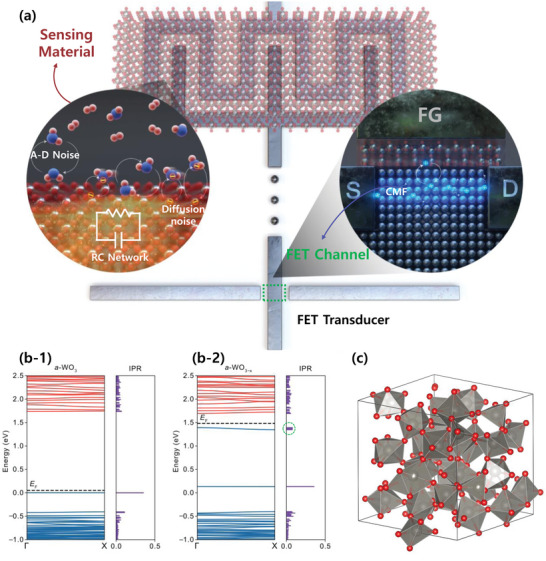
a) Schematic of the noise‐generation mechanism in the FET‐type gas sensor with a horizontal FG. In the sensing material, there are two noise sources: 1) Noise generated from the adsorption‐desorption (A‐D) of gas molecules to and from the sensing material, and 2) RC amplified shot noise generated from the carrier exchange between the adsorbed gas molecules and the sensing material. In the FET transducer, the carrier mobility fluctuation in the FET channel generates 1/*f* noise. The calculated band structures and corresponding inverse participation ratio (IPR) of the electronic states of b‐1) amorphous WO_3_ and b‐2) amorphous WO_3‐x_, respectively, are shown. The Fermi level (*E*_F_) is indicated by the dashed lines. c) Amorphous structure of WO_3_.

**Figure** **4**a shows a schematic illustration of the noise‐generation mechanism in the FET‐type gas sensor, showing the two independent noise sources of the FET channel and the sensing material. In the FET channel, carrier mobility scattering is the main noise source. In the sensing area, the shot noise caused by the local carrier change between the sensing material and gas molecules is amplified by the RC network of the sensing material. Therefore, the LFN characteristics of the FET‐type gas sensor are determined by the interplay between two different noise‐generation mechanisms. For a sensor with WO_3_ as the sensing material, the magnitude of the noise generated at the sensing material exceeds that in the FET channel. Therefore, the LFN of the sensor is determined by the fluctuation generated from the interaction between the gas molecules and WO_3_ not by carrier mobility scattering at the FET channel.

Here, it is important to reveal why the noise generated in the WO_3_ is greater than that in the FET channel, unlike indium oxide (In_2_O_3_)^[^ ^27^ ^]^ or IGZO^[^ ^42^, ^43^ ^]^ As previously reported, FET‐type gas sensors with In_2_O_3_ and IGZO show 1/*f* noise (Figure S5a (Supporting Information) and Ref. [42]), and the LFNs of the sensors are determined by the FET transducer (Figure S5b, Supporting Information). Such differences stem from the magnitude of the RC in the sensing material. In reducible metal oxide, depending on the energy level of the oxygen vacancy, the resistance of the reducible metal oxide can vary significantly.^[^ ^44^, ^45^ ^]^ An oxygen vacancy in metal oxides acts as an electron donor, thereby increasing the conductivity of *n*‐type metal oxides.^[^ ^44^ ^]^ In order to verify the properties of oxygen vacancy in WO_3_, a DFT analysis is conducted. Figure 4b(1,2) shows the calculated band structure and inverse participation ratio (IPR) of the electronic structure of amorphous WO_3_ and WO_3‐x_, respectively. The corresponding amorphous WO_3_ is presented in Figure 4c. In both WO_3_ and WO_3‐x_, the valence band maximum (VBM) and conduction band minimum (CBM) have small dispersions. Furthermore, as can be seen from the high IPR value, the states are localized. Even in the presence of an oxygen vacancy, high IPR values can be found near the CBM. It is verified that the oxygen vacancies in WO_3_ serve as a deep donor, which can be confirmed by the clustered IPR denoted by the dashed circle in Figure 4b‐2. The band structures and IPR values of different amorphous structures and oxygen vacancy positions are shown in Figure S6 (Supporting Information). Regardless of the amorphous structure and oxygen vacancy position, the oxygen vacancy in WO_3_ acts as a deep donor. For comparison, the band structures and IPR values of amorphous In_2_O_3_ are also calculated, as shown in Figure S7 (Supporting Information). Contrary to WO_3_, the oxygen vacancy in In_2_O_3_ serves as a shallow trap, resulting in a small resistance value of the film. Additionally, we investigate the LFN characteristics of the FET‐type gas sensor with a V_2_O_5_ sensing layer. Given that the V_2_O_5_ has much greater resistance than WO_3_, the *f*_c_ value of the FET‐type gas sensor is even <2 Hz, as shown in Figure S8a (Supporting Information). Figure S8b,c (Supporting Information) shows the *S*_VG_ and *f*  × *S*_VG_ of the sensor with V_2_O_5_ as a parameter of *T*.

From these results, the optimal material condition suitable for LFN spectroscopy can be provided. On the one hand, when the resistance of the metal oxide is too small (as in In_2_O_3_ and IGZO) the LFN of the sensor is determined by the FET channel. In this case, the LFN of the sensor is identical regardless of the type of gas reaction, thereby making it impossible to use LFN as a sensing feature for selective detection. On the other hand, when the resistance is too high (as in V_2_O_5_), the *f*_c_ is too small, necessitating a lengthy time for signal acquisition and preventing the rapid detection of the target gas. Therefore, it is important to select a sensing material with a magnitude of resistance that is neither too small nor large. From this perspective, WO_3_ with a deep level oxygen vacancy is the most suitable material for selective detection in FET‐type gas sensors.

### 2.4 Selective Gas Detection via LFN Spectroscopy

**Figure 5:**
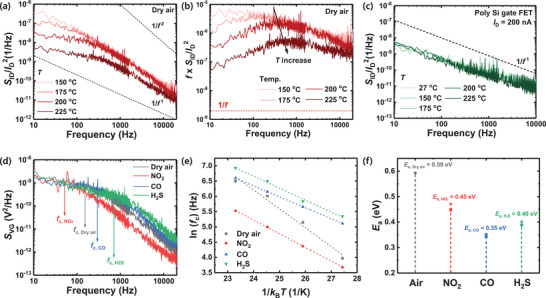
a) *S*_ID_/*I*_D_^2^ and b) *f*  × *S*_ID_/*I*_D_^2^ of the FET‐type gas sensor as a parameter of *T*. The PSDs are measured with *I*_D_ = 400 nA. c) *S*_ID_/*I*_D_^2^ of the poly‐Si gate FET versus *f* as a parameter of *T*. d) *S*_VG_ of the FET‐type gas sensor under different ambient conditions with dry air as a reference, NO_2_, H_2_S, and CO gases. e) Arrhenius plot (ln(*f*_c_) versus 1/*k*_B_ *T*) of the sensor under ambient dry air, NO_2_, H_2_S, and CO ambiences. d) *E*_a_ of the dry air, NO_2_, CO, H_2_S gases. The *E*_a_s are obtained at four different gas concentrations in each target gas. different *E*_a_ values for different gases.

At this stage, we investigate the effects of the gas reaction on the LFN characteristics of the FET‐type gas sensor. Because the LFN characteristics of the FET‐type gas sensor with WO_3_ are determined by the sensing material, exposure to different target gases would result in different LFN characteristics, which can be utilized to determine the selectivity of the sensor. For metal oxide sensing materials to react with the target gases, a *T* value that exceeds 100 °C is generally required. Accordingly, the effects of *T* on the LFN characteristics of the sensors are investigated. In addition, an investigation of the effects of *T* can confirm the validity of the proposed noise model. **Figure** **5**a shows the *S*_ID_/*I*_D_^2^ of the FET‐type gas sensor as a parameter of *T*. Note that the PSDs are measured at an *I*_D_ value of 400 nA. Here, *S*_ID_/*I*_D_^2^ multiplied by the frequency (*f* ×  *S*_ID_/*I*_D_^2^) versus the frequency is plotted in Figure 5b, allowing the separation of the Lorentzian‐like feature from the 1/*f* background. The maximum value of *f*  ×  *S*_ID_/*I*_D_^2^ corresponding to the *f*_c_ shifts to a higher frequency with increasing *T*. The increase of the *f*_c_ can be explained by the decrease of *R*_S_ with increasing *T*. In semiconducting metal oxide, the resistance is decreased with increasing *T* due to the increased carrier concentration and decreased potential barrier height at the grain boundaries. Figure 5c shows the *S*_ID_/*I*_D_^2^ of the poly‐Si gate FET versus *f* as a parameter of *T*. In contrast to the FET‐type gas sensors, the poly‐Si gate FET shows 1/*f* noise behavior regardless of how *T* changes. These results further demonstrate that the LFN characteristics of the sensor are not determined by the FET transducer but by the sensing material.

Figure 5d shows the *S*_VG_ of the FET‐type gas sensor under different ambient conditions, in this case dry air as a reference and, 500 ppb of NO_2_, 25 ppm of H_2_S, and 100 ppm of CO gases. The PSDs are measured after the response is saturated at 200 °C. Figure S9a (Supporting Information) shows *f*  × *S*_VG_ multiplied by a constant to distinguish the *f*_c_ clearly under different target gases. After the sensor is exposed to different types of target gases, a change in *f*_c_ clearly observed can be used as a distinctive sensing feature to realize selective detection. This occurs because the exposure of WO_3_ to different gases results in a change in the RC network and, thus a change of *f*_c_. Figure S9b (Supporting Information) shows *S*_Vg,sensing_ of the sensor versus the frequency as a parameter of *T* under ambient H_2_S.

Because the characteristic time depends on *T* exponentially, the noise activation energy (*E*_a_) can be extracted from the slope of the Arrhenius plot (ln(*f*_c_) vs 1/*k*_B_ *T*) in Figure 5e. The characteristic time of the charge fluctuation is affected by the gas sensing kinetics, resulting in The *E*_a_ value for each target gas is shown in Figure 5f, computed by taking the slope of the plot between *f*_c_ versus 1//*k*_B_ *T*. Note that the *E*_a_s are extracted at four different gas concentrations for each target gas (NO_2_: 100, 200, 300, and 400 ppb; H_2_S: 25, 50, 75, and 100 ppm; CO: 100, 200, 300, and 400 ppm). Figure S10 (Supporting Information) shows the Arrhenius plot (ln(*f*_c_) vs 1/*k*_B_ *T*) of the sensor under different concentrations of each target gas. Regardless of the change in the gas concentration, the *E*_a_ values of each of the target gases do not differ, demonstrating the validity of LFN spectroscopy for selective detection. The *E*_a_ values evaluated from the Arrhenius plot are 0.59, 0.45, 0.35, and 0.40 eV for the O_2_, NO_2_, CO, and H_2_S gases, respectively. It is important to note that the *E*_a_ values are clearly distinguishable such that the target gases can be completely separated. A unique *E*_a_ obtained from each target gas provides a physical basis for selective detection using LFN spectroscopy.

Without fabricating an external RC network, the RC network in the sensing material inherent to FET‐type gas sensor whose characteristics are sensitive to the change in gas ambient is used to achieve selectivity of the sensor. Therefore, the proposed method is more area‐ and cost‐efficient than other methods that implement the RC network at the circuit level. Furthermore, considering that *f*_c_ of PSD is affected by both *R*_S_ and *C*_S_, LFN spectroscopy has the advantages of selectively detecting a target gas in a mixed gas condition.

### 2.5 In‐Memory‐Computed FFT for an Area‐ and Energy‐Efficient Gas Sensing System

In order to realize LFN spectroscopy for selective detection, the Fourier Transform is required to calculate the PSD of the output sensing signal. In general, the Fourier Transform requires a large area and considerable energy for its computation, mainly due to the large amount of MAC operations. The computation burden generated by the separate computing and memory units can be significantly reduced by using an IMC. Conventional Discrete Fourier Transform (DFT) can easily be implemented in an IMC operation due to its simple vector‐matrix multiplication. However, high computational complexity of *O*(*n* · *n*) remains, increasing the demand for an efficient FFT method with computational complexity of *O*(*n* · *log* *n*). In this work, we adopt an in‐memory‐computed FFT with 4‐bit/cell 3D V‐NAND to improve the computational complexity, area, and energy efficiency factors.

The discrete Fourier Transform (DFT) is defined as follows:

(10) $$X_{k}=\sum_{n=0}^{N-1}x_{n}\cdot e^{-i\;2\pi kn/N}$$

Using Cooley and Tukey's Fast Fourier Transform (FFT), the DFT can be divided into two parts:^[^ ^46^ ^]^

(11) $$\begin{array}{ccc} X_{k} & = & \sum_{n=0}^{N-1}x_{n}\cdot e^{-i\frac{2\pi kn}{N}}\\ & = & \sum_{m=0}^{N/2-1}x_{2m}\cdot e^{-i\frac{2\pi k\left(2m\right)}{N}}+\sum_{m=0}^{N/2-1}x_{2m+1}\cdot e^{-i\frac{2\pi k\left(2m+1\right)}{N}}\\ & = & \sum_{m=0}^{N/2-1}x_{2m}\cdot e^{-i\frac{2\pi km}{\left(N/2\right)}}+e^{-i\frac{2\pi k}{N}}\sum_{m=0}^{N/2-1}x_{2m+1}\cdot e^{-i\frac{2\pi km}{\left(N/2\right)}} \end{array}$$

The first and second parts of Equation 11 represent the even part (*x*_2*m*_) and odd part (*x*_2*m* + 1_) of the input vector, respectively. By separating *N* size DFT into *N/*2 size of even part (*X*_*even*, *N*/2_) and the odd part (*X*_*odd*, *N*/2_), we can calculate DFT with size *N* (*O*(*N* ^2^)) from two DFTs with size *N*/2 (*O*(*N*/2^2^)). By dividing the *N*‐size DFT until the size of the part reaches a value of 1, *O*(*N* ^2^) complexity is reduced to *O*(*NlogN*). To calculate *X_N_*, we have to get values of *X*_*even*, *N*/2_ and *X*_*odd*, *N*/2_, which requires value of *X*_*even*, *N*/4_ … and so on. Recursive access of values of even part and odd part is required. A schematic description of recursive access of even and odd part at *N* = 8 is provided in Figure S11 (Supporting Information). Due to this procedure, the entire calculation is composed of log _2_ *N* cycles where the input vector is rearranged and multiplied by different FFT vectors in each cycle.

Before the calculation, the FFT vectors to be multiplied are split into four parts: +R_FFT_, −R_FFT_, +I_FFT_, −I_FFT_. Absolute values of the four split parts are converted into hexadecimal form, transformed to conductance representing each hex value, and mapped into NAND flash cells in the corresponding word‐line (WL).^[^ ^47^ ^]^ For each cycle, a read voltage (*V*_read_) is applied to the WL representing the FFT vector of the cycle, whereas pass voltage *V*_pass_ is applied to the other WLs. Because the conductance of NAND flash is much higher in the saturation region than near the *V*_th_ region, the conductance of NAND flash string can be dominated by the cell of the selected WL by setting *V*_read_ to be similar to *V*_th_ and *V*_pass_ to be higher than *V*_th_. This enables single‐row selection every cycle.

To perform the multiplication of the complex values in hardware, the input vectors are divided into four parts as with the FFT vectors: +R_input_, −R_input_, +I_input_, and −I_input_. During the calculation, the divided parts of the input vector and the FFT vector are multiplied and transferred to the appropriate parts of input vector of the next cycle. For example, results from +R_input_  ×  +R_FFT_, −R_input_  × −R_FFT_, −I_input_  × +I_FFT_, and I_input_  × −I_FFT_ are summed and passed to +R_input_ of the next cycle.^[^ ^48^ ^]^ A schematic description of the complex number multiplication process is provided in Figure S12 (Supporting Information).

**Figure 6:**
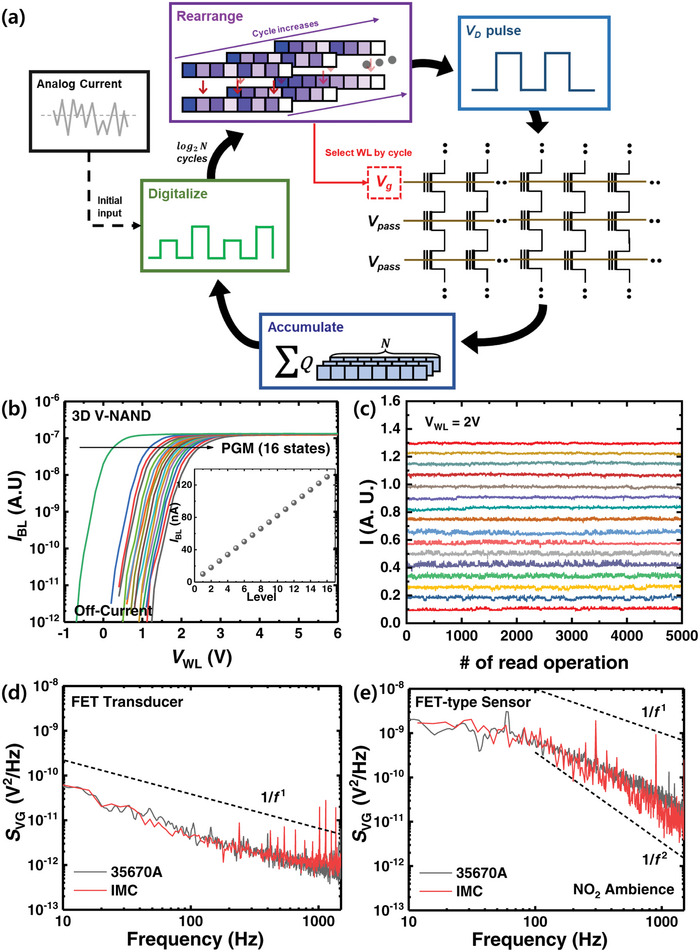
a) Schematic diagram of the in‐memory‐computing system of FFT using 3D V‐NAND. b) *I*_BL_‐*V*_WL_ diagram of 16 differently programmed flash cells. c) Current fluctuation versus the number of read operations of the 16 differently programmed flash cells. Comparison between the PSD obtained from the 35670A device and IMC‐based FFT in d) a FET transducer and e) a FET‐type gas sensor under ambient NO_2_. In both cases, the PSD obtained from the IMC‐based FFT shows identical results compared to that from the 35670A device, guaranteeing the validity of the proposed system.

**Figure** **6**a shows a schematic diagram explaining the IMC operation using 3D V‐NAND. Each cycle of the IMC operation consists of the following steps. 1) At the beginning of each cycle, odd and even indices of the signal vector are separated and rearranged. 2) After the rearrangement, the signal is divided into four parts, +R, −R, +I, and −I, transformed to binary form and then converted to the drain voltage pulses (*V_D_*), in each case. 3) The resulting currents from the source line (SL) are accumulated. 4) The accumulated currents are digitalized and transferred to the appropriate parts of the new input vector of the following

Figure 6b shows the *I*_BL_‐*V*_WL_ of a single cell composing the 3D V‐NAND with 16 different programmed levels. The channel conductance of the cell is modulated by a program operation via Fowler‐Nordheim (FN) tunneling. The 16 different states can be successfully modulated in this way (inset of Figure 6b). Figure 6c shows the current fluctuation of 16 differently programmed flash cells raised from the continuous read operation. During 5000 read operations, there is no overlap of the current between adjacent current levels (as determined by *V*_th_) in the flash cell, indicating that the read variation in each state does not affect the performance of the FFT. In order to verify the validity of the IMC FFT‐based 3D V‐NAND, we compare the PSDs of the FET transducer and the FET‐type gas sensors as obtained from a 35670A (commercially available equipment) and the proposed IMC‐based FFT. As shown in Figure 6d,e, the PSDs obtained from IMC‐based FFT show results identical to those from the 35670A device. The process of realizing LFN spectroscopy through in‐memory‐computing can be realized at a very fast speed. Because the corner frequency of the sensor under gas ambient conditions ranges from 10 to 10^3^ Hz, the maximum time required for the signal acquisition is <1 s if the ten times of signal sampling is used. After the signal is fed into 3D NAND array for detection, the processing time of FFT computation is very fast. According to our simulation results, the data processing time and computation time take ≈1 s which guarantees the real‐time detection. The data processing time may vary slightly by the performance of the computer, but it will fall below 1 s on modern computers with GHz clock frequency. The results demonstrate that the 3D V‐NAND can be successfully integrated into the gas sensing system for LFN spectroscopy to realize selective detection.

## 3 Conclusion

Here, we investigated the LFN characteristics of a FET‐type gas sensor with WO_3_ as a sensing material (connected to the FET‐type sensor's gate) and proposed the LFN spectroscopy to realize selective detection. The local charge fluctuation in the sensing material is amplified by a RC network, resulting in Lorentzian‐like behavior of the PSD. Fluctuations in the sensing material are transmitted to drain current fluctuations and added to the drain current noise stemming from the carrier mobility fluctuation in the FET channel. Because the LFN characteristics of the FET‐type gas sensor with the WO_3_ layer are determined by the sensing material, exposure to different target gases results in distinct LFN characteristics, which can be used to realize selective detection between NO_2_, CO, and H_2_S. Furthermore, it is verified through DFT calculations that WO_3_ is an optimal material for LFN spectroscopy owing to the deep‐level properties of the oxygen vacancy. Finally, an in‐memory‐computed FFT system is proposed based on 3D V‐NAND for an area‐ and energy‐efficient gas sensing system. The results are of great significance in that an entire system for selective detection using LFN spectroscopy is proposed, and a systematic analysis validates the solid physical basis of each component.

## 4 Experimental Section

### Gas Sensing and Electrical Measurement

A semiconductor parameter analyzer (B1500A) and a probe station with a test chamber, chuck, and gas inlet and output were used to assess the sensing performance capabilities of the sensors. The target gases were NO_2_, H_2_S, and CO, and the gas flow was regulated by a mass flow controller (MFC). Note that an external hot chuck raises the temperature required for the gas reaction. To adjust the gas concentration, the target gas was mixed with dry air with a relative humidity of 4% before being introduced into the test chamber.

The PSDs of the sensors were measured using a Keysight Semiconductor Device analyzer (B1500A), a Stanford Research Systems Low‐Noise Current Preamplifier (SR570), and a Keysight Dynamic Signal Analyzer (35670A). The measurement procedure was as follows: The B1500A device provides the voltage delivered to the CG. The output current flows to the SR570, which converts the current fluctuation into a voltage fluctuation. The dynamic signal from the SR570 was converted to a PSD via the 35670A device. The noise floor of the current amplifier in low noise mode was 4 ×  10^−27^ A^2^ Hz^−1^ (SR570 Manufacturer specs), significantly lower than the sensor noise. As a result, the PSDs measured in this work were clearly unaffected by the noise floor of the measurement system. Another factor to consider during the measurement was the spectral distortion of the PSD of the devices caused by the limited bandwidth of the SR570 device. The rated bandwidths in low noise mode were 2, 20, and 200 kHz with corresponding sensitivity levels of 100 nA, 1 µA, and 10 µA. Given the low‐frequency range in this experiment (*f* ≤  2  ×  10^4^ Hz), there would be no spectral distortion.

### Computational Details

All first‐principles calculations were conducted using the Vienna Ab initio Simulation Package (VASP).^[^ ^49^ ^]^ The Perdew‐Burke‐Ernzerhof (PBE) exchange‐correlation functional based on generalized gradient approximation (GGA) was implemented.^[^ ^50^, ^51^ ^]^ The cutoff energy for the plane‐wave basis set was set to 500 and 450 eV for the WO_3_ and In_2_O_3_ systems, respectively. For molecular dynamics in the WO_3_ system, the soft projector‐augmented wave pseudopotential was used, and the cutoff was reduced to 350 eV. Amorphous structures were generated with a conventional melt‐quench process. The initial WO_3_ amorphous cell contains 32 WO_3_ units (128 atoms in total), and the initial In_2_O_3_ amorphous cell contains 27 In_2_O_3_ units (135 atoms in total). First, atoms were put randomly into a cubic box with a density of 7.3 g cm^−3^. The melt‐quench process starts with the pre‐melting of the atoms at 4000 K for 5 ps, followed by 10 ps of melting at 2300 K for WO_3_ and 2800 K for In_2_O_3_. The structures were then quenched to 500 K at a constant cooling rate of –500 K ps^−1^. The final structures were fully relaxed with the force criteria of 0.02 eV Å^−1^. To generate the structures with an oxygen vacancy, one oxygen atom was removed from the relaxed pristine amorphous structure.

## Conflict of Interest

The authors declare no conflict of interest.

## Supporting information

Supporting Information

## Data Availability

The data that support the findings of this study are available in the Supporting Information of this article.
