# Supplementary material for: In‐Memory‐Computed Low‐Frequency Noise Spectroscopy for Selective Gas Detection Using a Reducible Metal Oxide
Source: Adv Sci (Weinh). 2023 Jan 16;10(7):2205725. doi: 10.1002/advs.202205725 (PMC9982552; doi:10.1002/advs.202205725)
Supplement: Supplementary file 1 — Supporting Information [file ADVS-10-2205725-s001.pdf]

ADVANCED SCIENCE
Open Access

Supporting Information

for *Adv. Sci.*, DOI 10.1002/advs.202205725

In-Memory-Computed Low-Frequency Noise Spectroscopy for Selective Gas Detection Using a Reducible Metal Oxide

*Wonjun Shin, Jaehyeon Kim, Gyuweon Jung, Suyeon Ju, Sung-Ho Park, Yujeong Jeong, Seongbin Hong, Ryun-Han Koo, Yeongheon Yang, Jae-Joon Kim, Seungwu Han and Jong-Ho Lee**

Supporting Information

In-memory-computed Low-frequency noise spectroscopy for selective gas detection using a reducible metal oxide

*Wonjun Shin, Jaehyeon Kim, Gyuweon Jung, Suyeon Ju, Sung-Ho Park, Yujeong Jeong, Seongbin Hong, Ryun-Han Koo, Yeongheon Yang, Jae-Joon Kim, Seungwu Han, and Jong-Ho Lee**

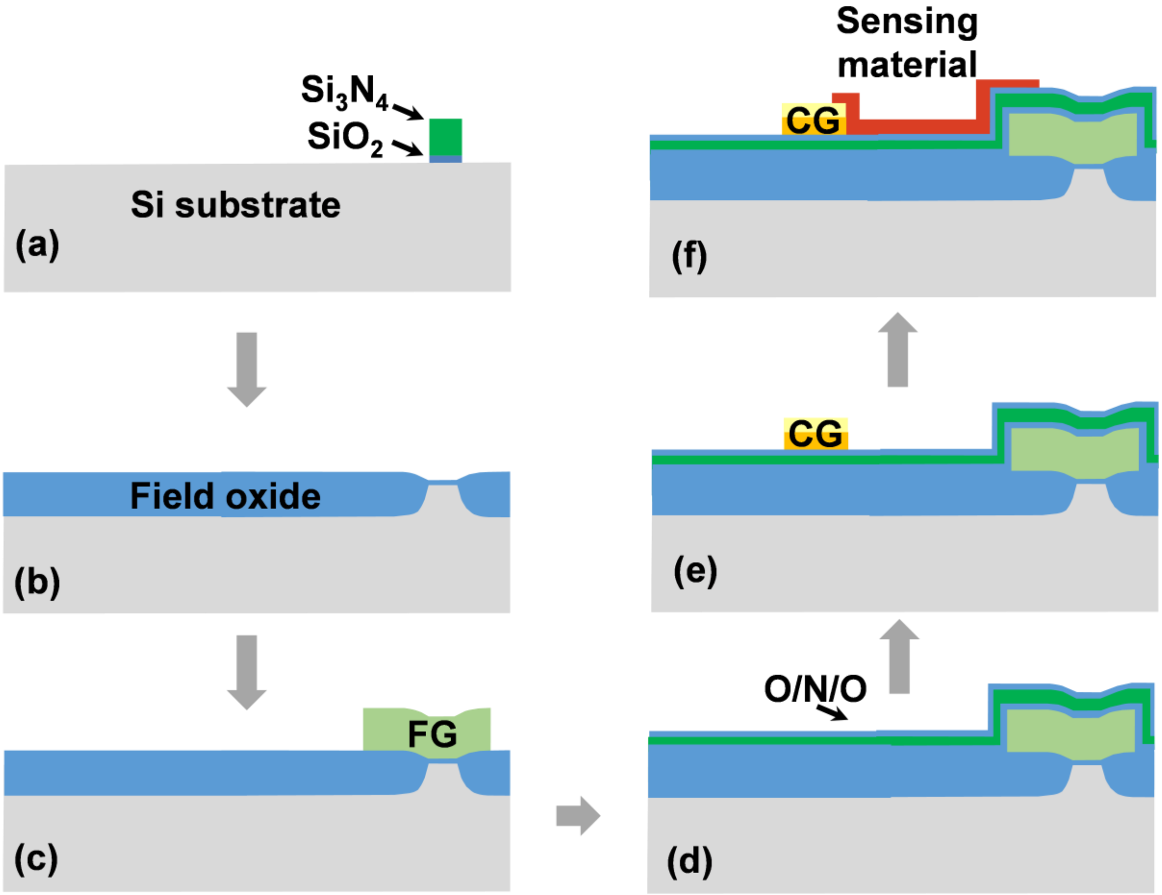

Figure S1. Schematic cross-sectional view of the key fabrication process for a horizontal FG FET-type gas sensor. On a 6-inch Si wafer, the gas sensors are produced utilizing CMOS technology: The flow of the fabrication process is as follows: (a) Active regions are defined by patterning a nitride layer deposited on a SiO_2 (pad oxide) layer grown on an *n*-type Si substrate. (b) An oxide with a thickness of 550 nm is then formed, and the nitride is removed. The pad oxide is eliminated, and a SiO_2 layer is produced as a sacrifice oxide. After performing ion implantation for threshold voltage control, the sacrificial layer is eliminated. Note that the channel implant for buried channel structure is conducted using ions whose dopant type is opposite to that of the substrate: BF^{2+} ions are implanted with implantation energy of 25 keV and dose of $2 \times 10^{12}/cm^2$. (c) Once a 10 nm thick gate oxide has been grown, n^+ polycrystalline Si is deposited to form the FG (equivalent gate electrode for normal FETs). (d) As a passivation layer (O/N/O), SiO_2 (10nm) / Si_3N_4 (20nm) / SiO_2 (10nm) is subsequently deposited. (e) After contact holes are defined, CG, source, and drain are produced. (f) The sensing layer is then deposited.

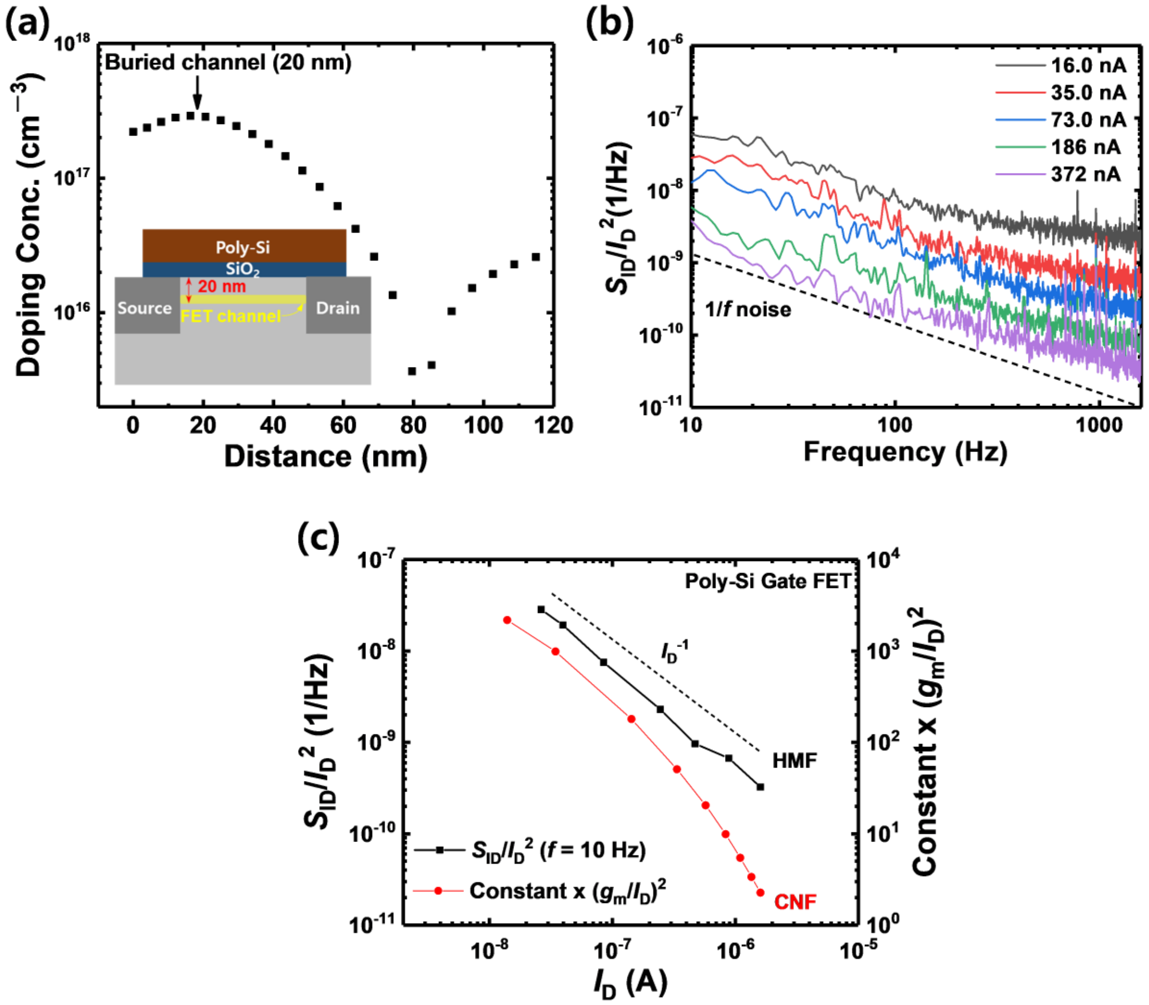

Figure S2. (a) Net doping profile near the channel surface obtained by TCAD simulation. The inset shows schematics of the FET channel in the poly-Si gate FET. (b) S_{ID}/I_D^2 of the poly-Si gate FET as a function of f at various bias conditions. (c) S_{ID}/I_D^2 and constant × $(g_m/I_D)^2$ versus I_D of the poly-Si gate FET.

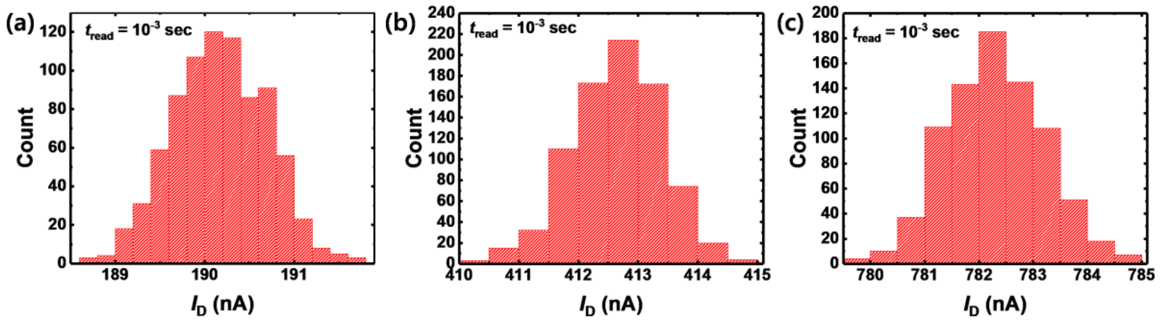

Figure S3. Gaussian distribution of three different I_D = (a) 190 nA, (b) 412 nA, and (c) 782 nA, respectively. Even though the PSD of the sensor shows a Lorentzian-like noise, the distribution of I_D follows a Gaussian function. From this, it is confirmed that the Lorenztzian-like noise does not stem from the RTN.

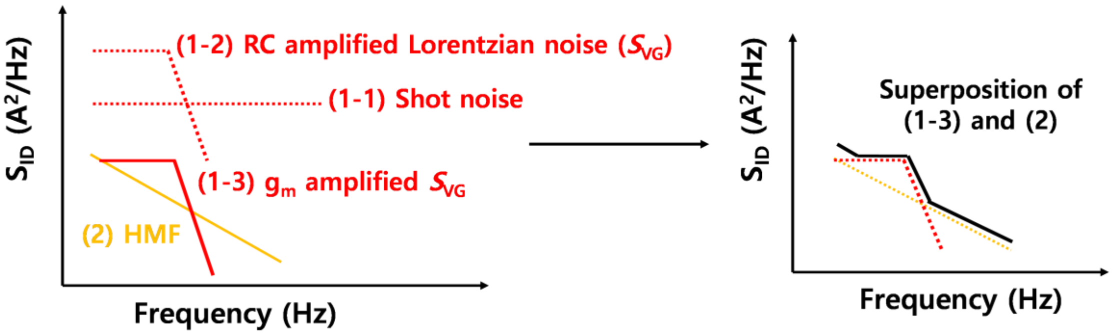

Figure S4. Schematic of noise generation mechanism in FET-type gas sensor with horizontal FG. In the FET channel, the 1/f noise is generated by the carrier mobility fluctuation in the buried channel. In the sensing material, the shot noise is amplified by the RC network and turned in to Lorentzian-like noise. Since two noise sources are independent, the superposition of each noise soucre results in a PSD of the FET-type gas sensor. The LFN characteristics of the sensor are determined depending on f_c and magnitude of Lorentzian-like noise.

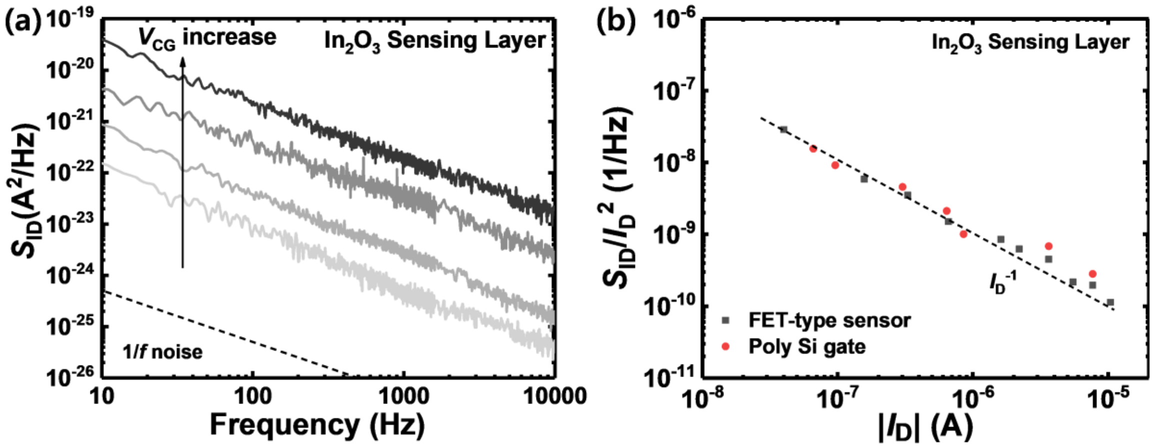

Figure S5. (a) S_{ID} of the FET-type gas sensor with horizontal FG having In_2O_3 as a sensing layer. (b) S_{ID}/I_D^2 versus I_D of the FET-type gas sensor with In_2O_3 and poly-Si gate FET. The LFN characteristics of the FET-type gas sensor are determined by the FET transducer when the In_2O_3 is used as a sensing layer.

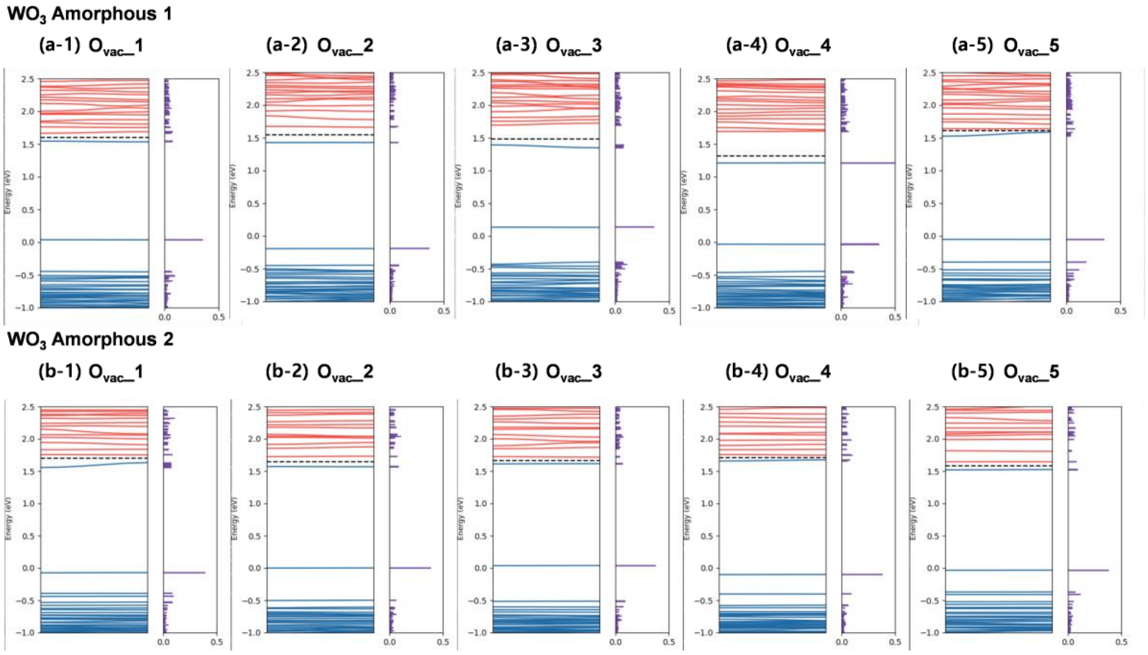

Figure S6. Two different amorphous structures are adopted in (a) and (b). Band structure and inverse participation ratio (IPR) of electronic structure of amorphous WO_3 having different position of oxygen vacancy are shown in each structure in (a-1)-(a-5) and (b-1)-(b-5), respectively.

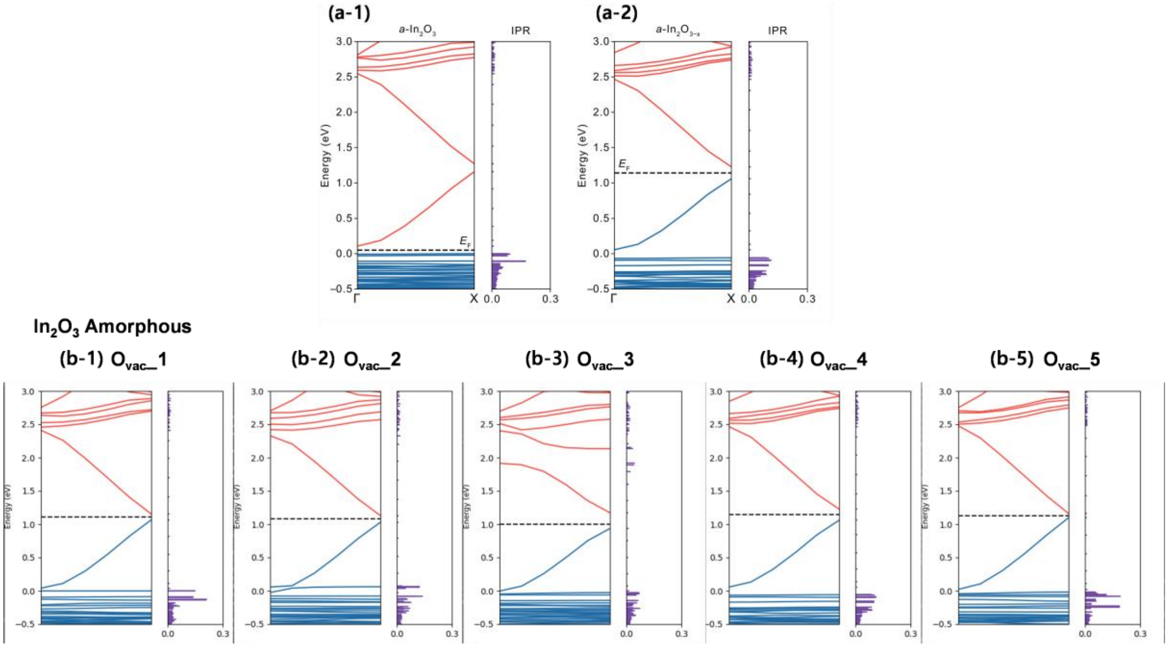

Figure S7. Calculated band structures and corresponding inverse participation ratio (IPR) of electronic states of (a-1) amorphous In_2O_3 and (a-2) amorphous In_2O_{3-x}, respectively. Band structure and IPR of electronic structure of amorphous In_2O_3 having different position of oxygen vacancy are shown in each structure in (b-1)-(b-5).

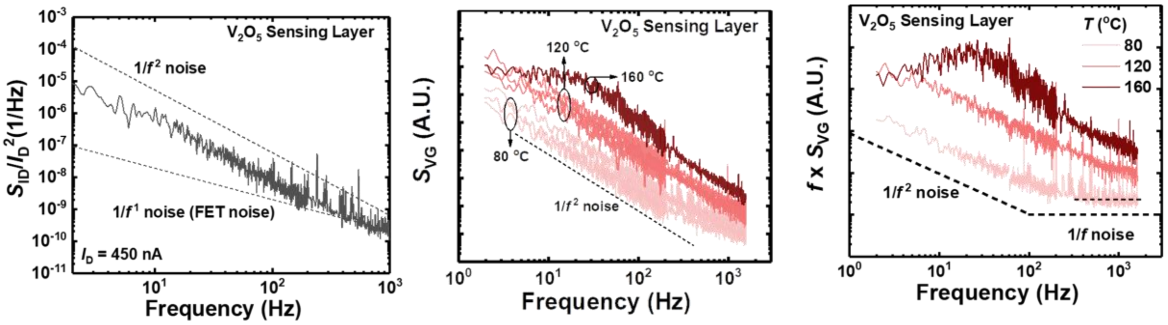

Figure S8. S_{ID} of FET-type gas sensor with horizontal FG having V_2O_5 as a sensing layer. The measured frequency ranges from 2 Hz to 10^3 Hz. The sensor with V_2O_5 has a much smaller f_c due to the large resistance of film. (b) S_{VG} and (c) $f \times S_{VG}$ of the sensor with V_2O_5 as a parameter of T, respectively.

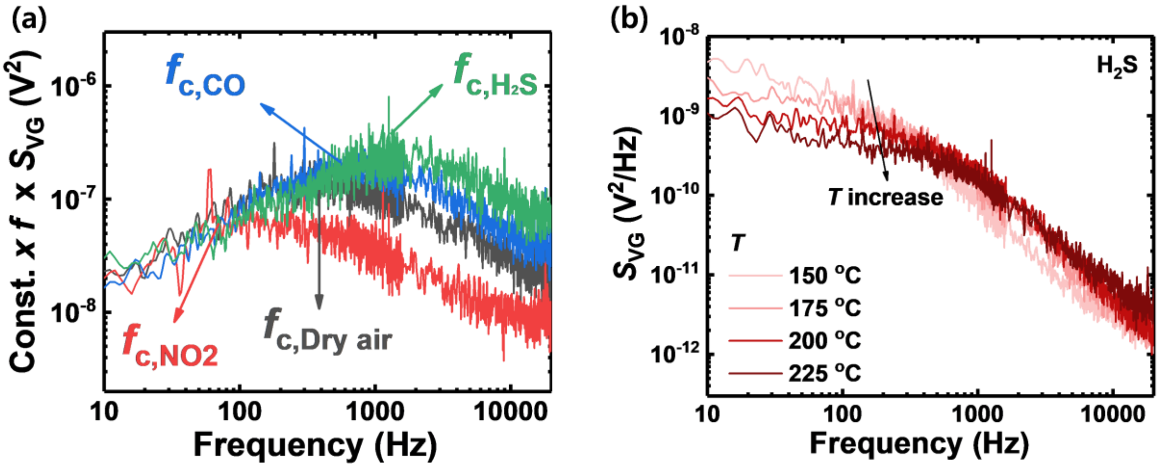

Figure S9. (a) $f \times S_{VG}$ of the sensor under dry air, NO_2, CO, and H_2S. The sensor has different f_cs depending on the target gas. Thus, the fc can be used as a unique sensing feature for the selective detection. (b) S_{VG} of the sensor under H_2S gas as a parameter of T.

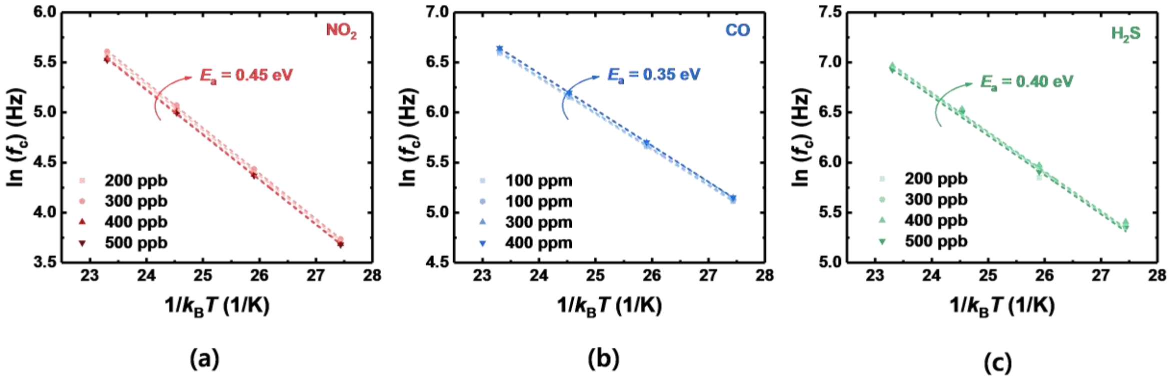

Figure S10. Arrhenius plot ($\ln(f_c)$ versus $1/k_BT$) of the sensor under different concentrations of (a) NO_2 (200, 300, 400, and 500 ppb)(b) H_2S (25, 50, 75, and 100 ppm), and (c) CO (100, 200, 300, and 400 ppm) ambiences, respectively.

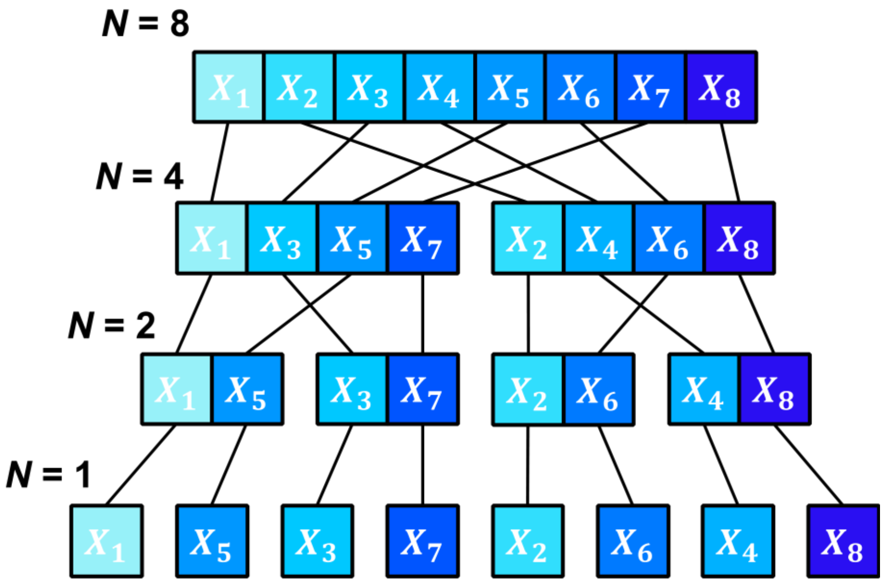

Figure S11. Schematic diagram of recursive access of even and odd part at $N = 8$.

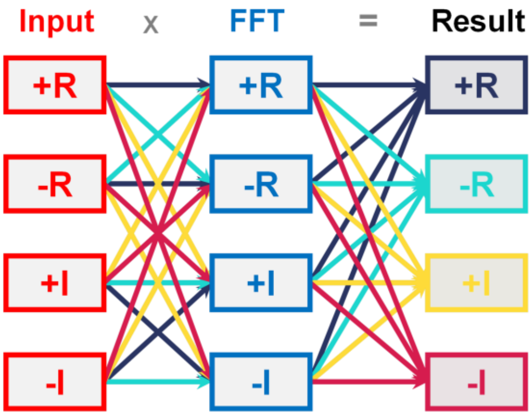

Figure S12. Schematic diagram to calculate complex number in 3D NAND flash array having different weights.
